# Inflammatory myofibroblastic tumor: A rare cause of invagination in adults

**DOI:** 10.12669/pjms.321.9326

**Published:** 2016

**Authors:** Remzi Kiziltan, Ozkan Yilmaz, Necat Almali, Caghan Peksen

**Affiliations:** 1 Remzi Kiziltanepartment of General Surgery, DursunOdabas Medical Center, School of Medicine, University of Yuzuncuyil, Van, Turkey; 2 Ozkan Yilmazepartment of General Surgery, DursunOdabas Medical Center, School of Medicine, University of Yuzuncuyil, Van, Turkey; 3 Necat Almali, Department of General Surgery, Training and Research Hospital, Van, Turkey; 4 Caghan Peksenepartment of General Surgery, DursunOdabas Medical Center, School of Medicine, University of Yuzuncuyil, Van, Turkey

**Keywords:** Inflammatory myofibroblastic tumor (IMT), Invagination, Adult, Ileus

## Abstract

Inflammatory myofibroblastic tumor (IMT) is a distinct pseudosarcomatous lesion arising in the soft tissues and interior organs of children and young adults. It is rarely seen in adults. It was first described in lungs. IMT can occur in any location in the body. However, it is seen most commonly in lungs, intestinal mesentery and liver. Non-mesenteric alimentary tract IMT’s are quite rare. The presented case is an ileal IMT that caused small bowel invagination. A 38 year-old male patient presented to the emergency department with the complaint of diffuse abdominal pain, distension and no passage of gas or stools for two days. An abdominal examination revealed distension and tenderness in the abdomen with no guarding or rebound tenderness. Computerized tomography (CT) of the abdomen was ordered. CT revealed an image compatible with invagination of the right lower quadrant of the abdomen and a mass inside the lumen measuring 4x3x3cm. The mass causing invagination was detected during the surgical operation. A segmentary small bowel resection and ileoileal anastomosis was performed. The patient was discharged uneventfully on the postoperative sixth day. The diagnosis of IMT was confirmed histologically and immunochemically.

## INTRODUCTION

Inflammatory myofibroblastic tumor (IMT) is a distinct pseudosarcomatous lesion arising in the soft tissues and interior organs of children and young adults. It is rarely seen in adults. Although it was first described in lungs,^1^ IMT can occur in any location of the body. However, it is most commonly seen in the lungs, intestinal mesentery, and liver. Histological specifications of IMT’s are chronic inflammatory cell infiltration and spindle cell proliferation.^2^,^3^ Its pathogenesis is unknown, although various allergic, immunological, and infectious mechanisms have been suggested in the etiology.^4^ Non-mesenteric alimentary tract IMT’s are quite rare. The presented case is an ileal IMT that caused small bowel invagination.

## CASE PRESENTATION

A 38 year-old male patient with no previous medical problem presented to the emergency department with the complaint of diffuse abdominal pain, distension, and no passage of gas or stools for two days. Abdominal examination revealed distension and tenderness in the abdomen with no guarding or rebound tenderness. His laboratory tests were as follows: WBC:12.1x10ˆ3/ml, urea:92.0 mg/dl, creatinine:1.15 mg/dl, chloride:111.0 mmol/L and glucose:127 mg/dl. Hemogram and other biochemical tests were within normal ranges. Upright direct roentgenogram of the abdomen revealed air-fluid levels of the small bowel type. Abdominal ultrasonography (US) disclosed a mass in the right lower quadrant of the abdomen and a computerized tomography (CT) of the abdomen was ordered. CT revealed an image compatible with invagination of the right lower quadrant of the abdomen and a mass inside the lumen measuring 4x3x3 cm. An ileo-ileal invagination was observed during laparotomy at a location 220 cm distal to the Treitz ligament. Following the correction of the invagination a solid mass with regular contours was noticed inside the lumen. A segmentary small bowel resection and ileo-ileal anastomosis was performed. The patient passed gas on the postoperative first day and he was fed on the third day. The patient was discharged uneventfully on the postoperative sixth day. The diagnosis of IMT was confirmed histologically and immunochemically.

**Fig.1:**
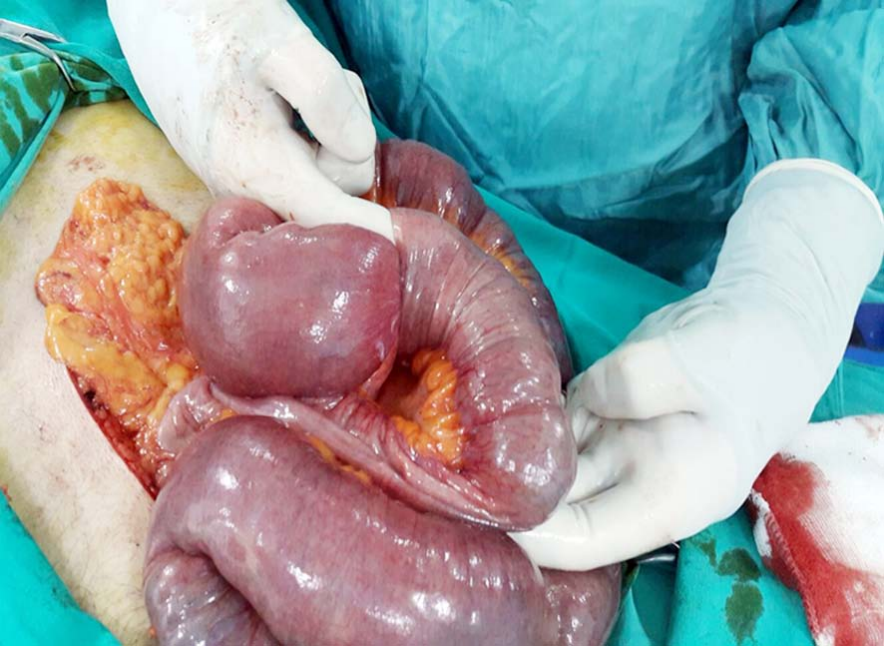
Intraoperative image.

**Fig.2:**
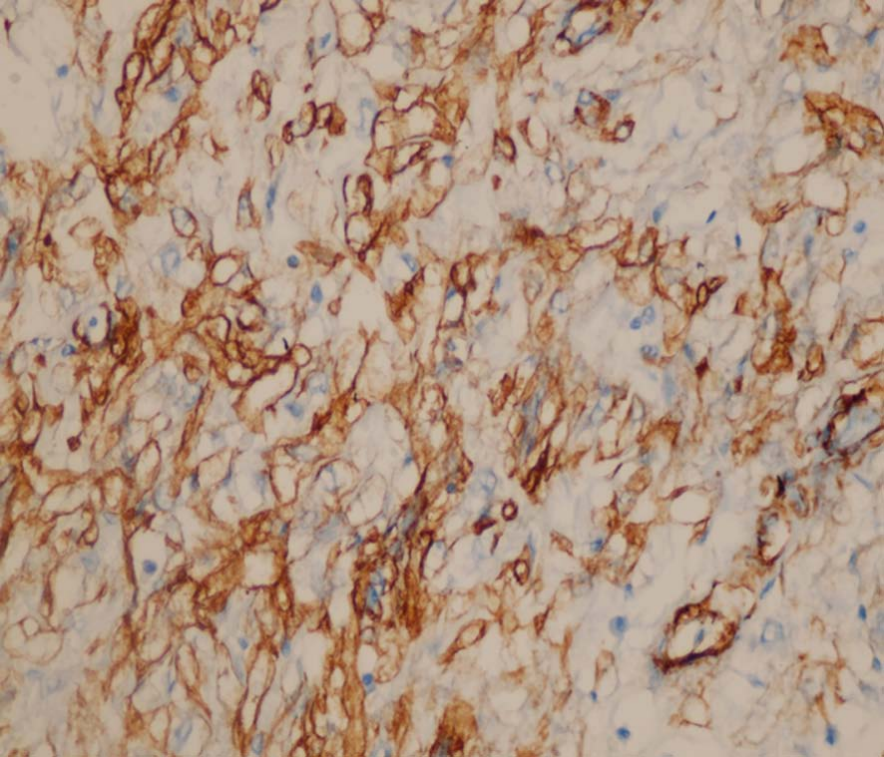
The expression of smooth muscle actin by immunohistochemistry in neoplastic cells (anti-smooth muscle actin ×400).

## DISCUSSION

This was a 38 year-old male patient who was diagnosed to have a small bowel IMT. IMT is seen more frequently in children and young adults and is more common in the female gender.^1^,^2^,^5^ Due to its different histological structure, the tumor can be localized in different organs and soft tissue. It is encountered with nonspecific clinical symptoms and radiological findings, according to the localization of the tumor. IMT can be palpated when it is in the abdominal cavity and reaches to greater dimension. As in this case report, it can also cause nonspecific abdominal pain, weight loss, fever and small bowel obstruction, as well as invagination. It is usually encountered as an intramurally localized IMT which causes diarrhea and intestinal obstruction in children.^6^

IMT has typically been considered as a benign lesion. However, some variants of the tumor may be locally destructive. Biselli et al. reported that chromosomal aberrations may be present in IMT.^7^

Atypical intestinal gas distribution in the upright abdominal X-ray, and intratumoral calcifications, might be observed in cases with small bowel IMT. A vague solid mass with regular contours can generally be seen in abdominal ultrasonography and CT imaging. In this present case, a solid mass measuring 4 x 3 cm in the left lower quadrant of the abdomen, and an image compatible with invagination in the same area, was detected using abdominal tomography.

Microscopically, IMT resembles gastrointestinal stromal tumors. Tumor cells may invade the muscularis propria and even the adventitia without the presence of atypia or hyperchromatism.

Immunohistochemical analysis plays a major role in the definitive diagnosis of IMT. Tumor cells, characteristically vimentin positive and with no expression of CD117 and CD34, are present.^1^,^8^ The cells are positive for smooth muscle actin whether desmin expression or not S100 positivity is present.^9^ In the present case, immunohistochemical staining showed that actin (+), vimentin(+) and caldesmon (+) were found to be positive.

Surgical excision is the primary choice of treatment in cases with IMT.^10^ Following complete excision, the rate of local recurrence is less than 10%. Surgical excision with a negative surgical margin was performed in the case presented. Chemotherapy and radiotherapy was demonstrated to be ineffective in the literature for these tumors,^3^ nevertheless, combinations of radiotherapy, chemotherapy and non-steroidal anti-inflammatory drugs (NSAIDs) might be an alternative choice of treatment in unresectable cases of IMT.^11^
